# The ground state of the Kondo insulator

**DOI:** 10.1038/s41598-023-42697-8

**Published:** 2023-09-16

**Authors:** Igor N. Karnaukhov

**Affiliations:** https://ror.org/01a1qdv64 grid.435300.1 0000 0004 0482 7152 G.V. Kurdyumov Institute for Metal Physics, 36 Vernadsky Boulevard, Kyiv, 03142 Ukraine

**Keywords:** Physics, Condensed-matter physics, Magnetic properties and materials

## Abstract

The Kondo insulator (KI) is the state of an electron liquid in the Kondo lattice at half filling, studied within the mean field approach. We demonstrate, that the ${Z}_2$-field, which is formed by interaction between electrons and local moments, leads to an insulator state in a lattice with a double cell lattice. In the ground state, electrons and local moments form singlets; in this case, no spin or charge density waves are realized in a lattice with a double cell. The Majorana-type gap spectrum of the quasi-particle excitations is realized in the ${ Z}_2$ -field. The gap in the spectrum decreases with increasing external magnetic field; it closes at a critical value at the insulator-metal phase transition point. In metal phase the lattice remains with a double cell. Thus, the introduction of the ${ Z}_2$ -field allows us to answer the key question what is the ground state of KI.

## Introduction

In contrast of the Kondo problem, whose exact solution was obtained in the case of weak interaction in the continuum approximation^1,2^, the behavior of an electron liquid in a Kondo lattice is an unsolved problem in condensed matter physics. In the Kondo problem the scattering electrons by a local moment within a spin flip leads to the Abrikosov-Suhl resonance, a new behavior of an electron liquid at low temperatures and magnetic field.

Speaking of the Kondo lattice, we do not know the answers to simple but important questions like what is the ground state of an electron liquid in KI, and why is there a large Fermi surface at the conservation of the number of electrons, what is the nature of the charge and spin gaps in the excitation spectrum^3–10^. When solving the Kondo lattice problem, it is also necessary to take into account the scattering of electrons by local moments with spin flip (as it takes place in the Kondo problem). The effective Hamiltonian should also not break the symmetry of the model Hamiltonian. This is a non-trivial problem that has not yet been solved, so we cannot say anything definitive about what the ground state is implemented in KI.

However, despite this pessimistic introduction, the purpose of the article is to answer a main question, what is the ground state of the electron liquid in the Kondo lattice at half filling. The antiferromagnetic exchange interaction between electrons and local moments leads to ${Z}_2$-field, whose an uniform configuration forms a lattice with a double cell in KI. Note that charge or spin density waves are not realized in a lattice with a double cell; in this sense, this is an unusual state.

## Model

The Hamiltonian of the spin-$\frac{1}{2}$ Kondo lattice dimension D ${{{\mathscr {H}}}}={{{\mathscr {H}}}}_0+{{{\mathscr {H}}}}_{K}$ includes two terms, the first of which is determined by energy of electrons, the second one is determined by the contact exchange interaction of these electrons with local moments when they are arranged regularly

1 $$\begin{aligned}{} & {} {{{\mathscr {H}}}}_0= - \sum _{<i,j>}\sum _{\sigma =\uparrow ,\downarrow }c^\dagger _{i \sigma } c_{j \sigma }-2h \sum _{j}(s^z_j+S^z_j), \nonumber \\{} & {} {{{\mathscr {H}}}}_K= 2\sum _{j=1}^N [J s^z_jS^z_j+K (s^x_jS^x_j+s^y_jS^y_j)], \end{aligned}$$

where $c^\dagger _{j \sigma }$ and $c_{j \sigma }$ are the fermion operators determined on a lattice site *j*, $\sigma =\uparrow ,\downarrow$ denotes the spin of electron, the hopping integral between the nearest-neighbor lattice sites is equal to one, the spin operators of electrons $s_j^{\alpha }=\frac{1}{2}c^\dagger _{j \sigma }\sigma ^{\alpha }_{\sigma \sigma '}c_{j\sigma '}$ are determined by the Pauli matrices $\sigma ^{\alpha }$ ($\alpha =x,y,z$), ${{\textbf {S}}}_j$ is the spin-$\frac{1}{2}$ operator defined on the lattice site *j* (*S* is its value), $J\ge 0$ and $K>0$ are the magnitudes of the exchange interaction ($K=J>0$ corresponds to an isotropic antiferromagnetic exchange interaction, $J=0, K>0$ corresponds to a strong anisotropic interaction), *h* is an external magnetic field ($g-$ factor is 2, we assume the Bohr magneton is 1), N is the total number of lattice sites.

We study that the behavior of an electron liquid in the chain (1D) and on the square (2D) and cubic (3D) lattices at half-filling.

## The ground-state of an electron liquid

We rewrite the term of the Hamiltonian (1) ${{{\mathscr {H}}}}_{K}$ in detail in the following form

2 $$\begin{aligned}{} & {} {{{\mathscr {H}}}}_K= \sum _{j=1}^N[2J s^z_jS^z_j+K(s^+_jS^-_j + s^-_jS^+_j)], \end{aligned}$$

where spin operators are redefined in the terms of fermionic operators, $s^z_j=\frac{1}{2}(m_{j\uparrow }-m_{j\downarrow })$, $s^+_j=c^\dagger _{j \uparrow } c_{j\downarrow }$, $s^-_j=c^\dagger _{j \downarrow } c_{j\uparrow }$, here $m_{j\sigma }=c^\dagger _{j \sigma } c_{j \sigma }$, $m_{j}=m_{j\uparrow }+m_{j\downarrow }$ are the density operators.

We use the following presentation for the ${{{\mathscr {H}}}}_{K}$ term: $2Js^z_jS^z_j+K(s^+_jS^-_j + s^-_jS^+_j)=-J (s^z_j-S^z_j)^2-K(s^+_j-S^+_j)^\dagger (s^+_j-S^+_j) +(J-K) (S^z_j)^2-\frac{1}{4}(J+2 K)m_{j}^2+\frac{1}{2}(J+K)m_j+K S(S+1) \Longrightarrow 2\Lambda _{j} (s^z_j-S^z_j)+\lambda _{j}(c^+_{j\uparrow }c_{j\downarrow }-S^+_{j})+\lambda ^*_{j}(c^+_{j\downarrow }c_{j\uparrow }- S^-_{j} )+2\mu _{j} m_{j}$. We will study in detail the case of $S=\frac{1}{2}$. $(S^z_j)^2$ -operator is conserved, $\mu$-component shifts the Fermi energy, so it can be neglected. Using the Hubbard-Stratonovich transformation we introduce the effective Hamiltonian which is determined by two component ${Z}_2$-field. The canonical functional is determined by the action, which follows from the Hubbard-Stratonovich transformation

3 $$\begin{aligned}{} & {} {{{\mathscr {S}}}}=\sum _{j} \frac{\Lambda _j^2}{J}+\sum _{j} \frac{|\lambda _j|^2}{K}+\int _0^\beta d\tau \sum _{{\textbf {k}}}\Psi _{{\textbf {k}}}^\dagger (\tau )[\partial _\tau + {\mathscr {H}}_{eff}({{\textbf {k}}})]\Psi _{{\textbf {k}}} (\tau ), \end{aligned}$$

where $\Psi _{{\textbf {k}}} (\tau )$ is the wave function, $\beta =\frac{1}{k_B T}$, $k_B$ is the Boltzmann constant, T is temperature, ${{\textbf {k}}}=(k_x,k_y,k_z)$ is the wave vector.

The effective Hamiltonian ${{{\mathscr {H}}}}_{eff}$ determines the ground state of the model and low-energy excitations at half filling occupation. We expect that $\Lambda _{{{\textbf {j}}} }$ and $\lambda _{{{\textbf {j}}} }$ are independent of time because of translational invariance. We study the ground state of the Kondo insulator, the low temperature hehavior of an electon liquid is not studied. and low-energy excitations corresponding to fluctuations of the saddle point solution are not taken into account.

We can define an effective Hamiltonian ${{{\mathscr {H}}}}_{eff}$, which describes the behavior of the electron liquid in the Kondo lattice in the mean field approach ${{{\mathscr {H}}}}_{eff}={{{\mathscr {H}}}}_0+ \sum _{j}[2\Lambda _{j} (s^z_j-S^z_j)+\lambda _{j}(c^+_{j\uparrow }c_{j\downarrow }-S^+_{j})+\lambda ^*_{j}(c^+_{j\downarrow }c_{j\uparrow }-S^-_{j})$.

Let us consider the equations for the one-particle wave functions $\psi ({{\textbf {j}}},\sigma )c^\dagger _{{{\textbf {j}}} \sigma }\phi ({{\textbf {j}}},\pm \sigma )S^{\pm }_{{{\textbf {j}}} }$ ($\sigma =\uparrow ,\downarrow )$ with energy $\varepsilon$, the $\psi ({{\textbf {j}}},\sigma )$ and $\phi ({{\textbf {j}}},\sigma )$ amplitudes satisfy the following equations :

4 $$\begin{aligned}{} & {} (\varepsilon -\Lambda _{{{\textbf {j}}}})\psi ({{\textbf {j}}},\sigma ) +\lambda _{{{\textbf {j}}}} \psi ({{\textbf {j}}},-\sigma )+\sum _{{{\textbf {1}}}}\psi ({\textbf {j+1}},\sigma )=0, \nonumber \\{} & {} (\varepsilon + \Lambda _{{{\textbf {j}}}})\psi ({{\textbf {j}}},-\sigma )+\lambda ^*_{{{\textbf {j}}}}\psi ({{\textbf {j}}},\sigma )+ \sum _{{{\textbf {1}}}}\psi ({\textbf {j+1}},-\sigma )=0, \nonumber \\{} & {} (\varepsilon +\Lambda _{{{\textbf {j}}}})\phi ({{\textbf {j}}},\sigma ) -\lambda _{{{\textbf {j}}}} \phi ({{\textbf {j}}},-\sigma )=0,\nonumber \\{} & {} (\varepsilon -\Lambda _{{{\textbf {j}}}})\phi ({{\textbf {j}}},-\sigma ) -\lambda ^*_{{{\textbf {j}}}} \phi ({{\textbf {j}}},\sigma )=0, \end{aligned}$$

where sums over the nearest lattice sites. The real variables $\Lambda _{{{\textbf {j}}}}\rightarrow \pm \Lambda _{{{\textbf {j}}}}$ and $\lambda _{{{\textbf {j}}}}\rightarrow \pm \lambda _{{{\textbf {j}}}}$ are identified with a static two component ${Z}_2$- field determined on the lattice sites. A confuguration of this field, which corresponds to an energy minimum, defines the ground state. The local moments form the flat band states, with energies $\varepsilon _S=\pm \sqrt{\Lambda ^2+\lambda ^2}$, here $\Lambda _{{{\textbf {j}}}}^2=\Lambda ^2,|\lambda _{{{\textbf {j}}}}]^2=\lambda ^2$. The local moments are arranged regularly at the lattice sites, their energy does not depend on $S^z_{{{\textbf {j}}}}$.

In contrast to well known models^11,12^, where a free condiguration of the $Z_2$-field ($\lambda _j=\lambda$) corresponds to minimum of energy, an uniform sector with $\Lambda _{{{\textbf {j}}} }=-\Lambda _{{\textbf {j+1}}}=\Lambda$, $\lambda _{{{\textbf {j}}} }=-\lambda _{{\textbf {j+1}}}=\lambda$ corresponds to minimum of energy in the Kondo insulator^13,14^. This field configuration leads to the lattice with a double cell, does not break the translational symmetry. Detailed numerical analysis shows, that an uniform sector with $\Lambda _{{{\textbf {j}}} }=-\Lambda _{{\textbf {j+1}}}=\Lambda$, $\lambda _{{{\textbf {j}}}} =-\lambda _{{\textbf {j+1}}}=\lambda$ corresponds to the ground state of an electron liquid for arbitrary values of *J* and *K*. Using Eq. (4) we calculate the energies of the quasi-particle excitations wich correspond to this uniform configuration of the ${\mathbb {Z}}_2$-field. The spectrum includes two branches of local moments $\varepsilon _S$ and two branches of electrons $\varepsilon _s({{\textbf {k}}})=\pm \sqrt{\Lambda ^2+\lambda ^2+|w({{\textbf {k}}})|^2}$, here $w({{\textbf {k}}})=\sum _{\alpha }^D[1+\exp ( i k_\alpha )]$. The spectrum of the quasi-particle excitations is symmetric with respect to zero energy, has the Majorana type at half filling, the chemical potential is zero. Despite the fact that the effective Hamiltonian does not conserve the total spin, the wave function (4) at the same time conserve the total spin, since the flip of the electron spin is accompanied by the reverse flip for the local momentum located at the same lattice site. The values of the ${Z}_2$-field components satisfy the energy minimum or the saddle point of the action, self-consistent equations have the following form at $T=0K$

5 $$\begin{aligned}{} & {} \frac{2\Lambda }{J}=\frac{1}{N}\sum _{{{\textbf {k}}}}\frac{\Lambda }{|\varepsilon _s({{\textbf {k}}})|}+\frac{\Lambda }{|\varepsilon _S|},\nonumber \\{} & {} \frac{2\lambda }{K}=\frac{1}{N}\sum _{{{\textbf {k}}}}\frac{\lambda }{|\varepsilon _s({{\textbf {k}}})|}+\frac{\lambda }{|\varepsilon _S|}. \end{aligned}$$

where $\Lambda =\lambda \ne 0$ for isotropic $J=K>0$, and $\Lambda =0, \lambda \ne 0$ for anisotropic $J=0, K>0$ exchange interactions.

The behavior of the electron liquid in the case of strongly anisotropic, when $J=0$, $K>0$, and isotropic, when $J=K>0$, exchange antiferromagnetic interaction will be considered in detail.

**Figure 1:**
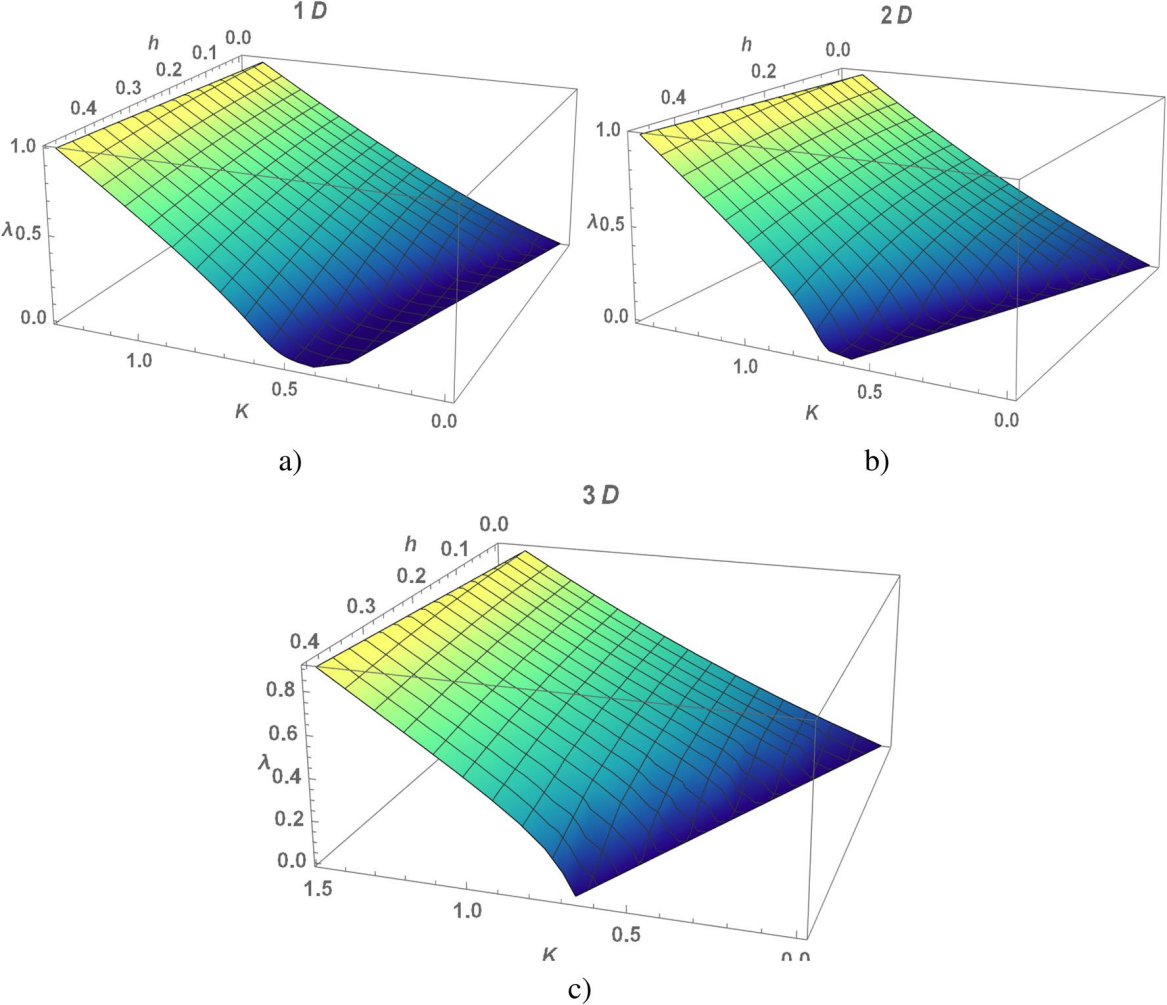
(Color online) $\lambda -$value as a function of the exchange integral *K* and magnetic field *h* , calculated for the chain (**a**), square (**b**) and cubic (**c**) lattices.

**Figure 2:**
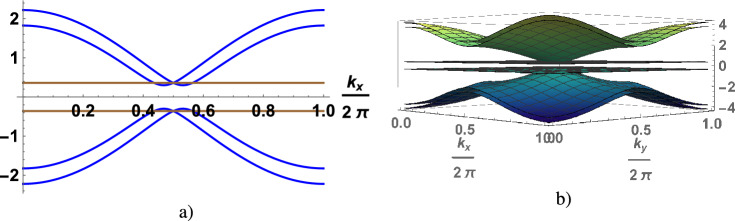
(Color online) The spectrum of quasi-particle excitations of electron liquid ( the gap in the spectrum of charge excitations is equal to $2\lambda$) in the chain (**a**) and square lattice (**b**) as a function of the wave vector, calculated for $\lambda =0.3$, $h=0.2$ ($K=0.524$ for chain and $K=0.597$ for square lqttice).

**Figure 3:**
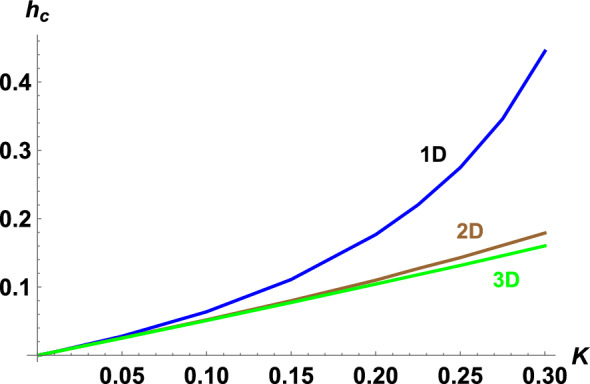
(Color online) Critical value of magnetic field $h_c$, at which the gap in the quasi-particle spectrum closes, as a function of the exchange integral, calculated for different dimension of the Kondo lattice.

**Figure 4:**
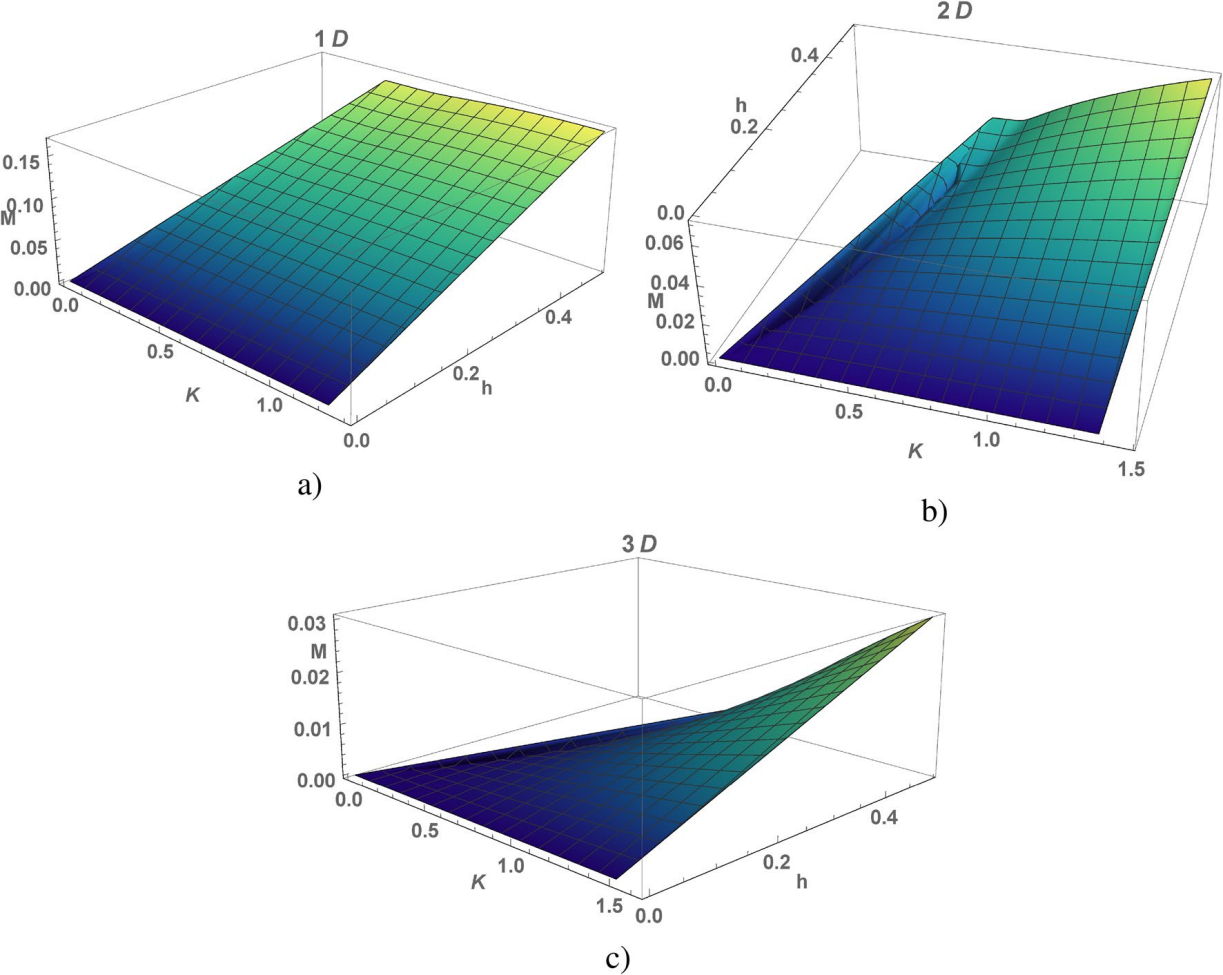
(Color online) Magnetization density as a function of the exchange integral *K* and magnetic field *h* , calculated for the chain (**a**), square (**b**) and cubic (**c**) lattices.

**Figure 5:**
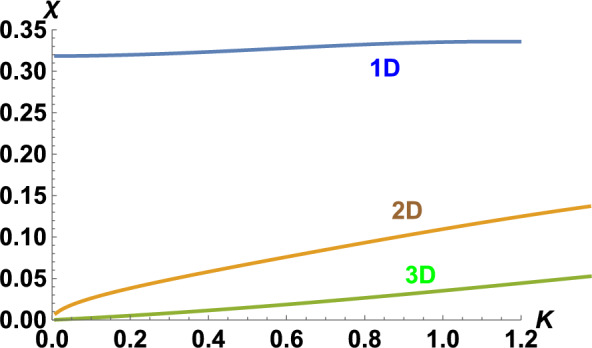
(Color online) Static magnetic susseptibility as a function of an exchange integral calculeted for different dimension of the model.

**Figure 6:**
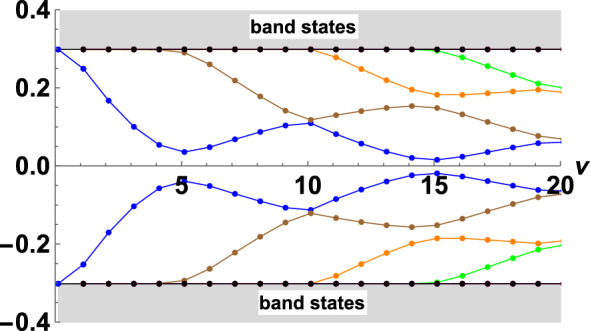
(Color online) The excitation energies corresponding to the $Z_2$-field configuration with $\nu$ “defects” are calculated for various $\nu$ (marked with dots, the lines correspond to the excitation branches). Calculations were carried out for a chain with $\lambda =0.3$, $h=0.1$, $K=0.476$.

### Strongly anisotropic exchange interaction $J=0$, $K>0$

For a strongly anisotropic exchange interaction, the ${Z}_2$- field is one-component, since $\Lambda =0$ and $\lambda \ne 0$. According to the numerical analysis, the solutions $\lambda _{{\textbf {j}}} =-\lambda _{\textbf {j+1}}=\lambda$ correspond to the minimum energy for arbitrary values of the exchange integral *K* and magnetic field *h*.

In magnetic field the energies of the quasi-particle excitations transform to $\varepsilon _{s,+}({{\textbf {k}}})=\pm \sqrt{\Lambda ^2+\lambda ^2+(h+|w({{\textbf {k}}})|)^2}$, $\varepsilon _{s,-}({{\textbf {k}}})=\pm \sqrt{\Lambda ^2+\lambda ^2+(h-|w({{\textbf {k}}})|)^2}$, $\varepsilon _S=\pm \sqrt{\Lambda ^2+\lambda ^2+h^2}$. The spectrum is symmetrical with respect to zero energy or chemical potential, which is zero at half filling for an arbitrary magnetic field.

In the electron spectrum the gap opens at $\lambda \ne 0$ and is equal to $2\lambda$ at $\Lambda =0$. According to Eq. (5) its value is determined by *K* and *h*. Using Eq. (5) we numerically calculate $\lambda$ as function of *K* and *h* for the chain (Fig 1a), square (Fig 1b) and cubic (Fig 1c) lattices. Should be note an universal behavior of an electron liquid in KI, the curves in Figs are similar for an arbitrary dimension. In a weak coupling limit at $K\rightarrow 0$ the last term in Eq. (5) dominates, so $\lambda \rightarrow \frac{K}{2}$. We ilustrate the spectrum of the quasi-particle excitations in Fig. 2a for the chain and Fig. 2b for the square lattice. The magnetic field breaks the spin degeneracy of the spectrum of the electrons, spreading the branches. The $\lambda$- value (or the value of the gap) decreases with increasing magnetic field. A critical value of the magnetic field $h_c$, at which the gap closes^15^, depends on $K-$value. Numerical calculations of $h_c$ are shown in the Fig. 3 (the curves are calculated for different dimension of the model). In magnetic field $h_c$ the phase transition from insulator to the metal states is realized, KI is stable at $h<h_c$. An uniform configuration of the ${Z}_2$-field corresponds to minimum energy in metal state with the local doubling of period of the original lattice, in other words the metal state is also realized in the lattice with a double cell.

Magnetic properties of an electron liquid in KI are determined by both band electrons and local moments, they are determined by the uniform configuration of the ${Z}_2$-field. Electrons and local moments form singlet states in the lattice with a double cell, which are not fixed in time. In absence of magnetic field the energies of the quasi-particle excitations degenerate in spin, so the magnetization density $M=\frac{2}{N}\sum _{j}(s^z_j+S^z_j)$ is zero. The magnetic field does not break this energy degeneracy for local moments, so the magnetization is given by an electron term $M=\frac{1}{N}\sum _{j}(m_{j,\uparrow }-m_{j,\downarrow })$:

6 $$\begin{aligned} M=\frac{h}{N}\sum _{{{\textbf {k}}}} \frac{1}{|\varepsilon _{s +}({{\textbf {k}}})| +|\varepsilon _{s,-}({{\textbf {k}}})|} (\frac{\lambda ^2 + h^2- |w({{\textbf {k}}})|^2 }{ |\varepsilon _{s,+}({{\textbf {k}}})\varepsilon _{s,-}({{\textbf {k}}})| } + 1 ). \end{aligned}$$

The calculations of the magnetization density *M* as function of magnetic field and the exchange integral *K* are presented in Fig. 4 for different dimension of the model. Formula for a static magnetic susceptibility leads from *M* at $h\rightarrow 0$ $\chi =\frac{1}{N}\sum _{{{\textbf {k}}}} \frac{\lambda ^2 }{(\lambda ^2 +| w({{\textbf {k}}})|)^{3/2}}$. The value of a static magnetic susceptibility is calculated as function of the exchange integral for different dimension of the model. The calculations are shown in Fig. 5, the susseptibility is a monotonic function of *K*.

An uniform configuration $\lambda _j=-\lambda _{j+1}$ stabilises the state with a double cell, a free configuration $\lambda _j=\lambda _{j+1}$ corresponds to gapless state on original latiice with higher energy. We define an unstable configuration of the $\lambda$-field with one “defect” as $-\lambda _{j-2}=\lambda _{j-1}=\lambda _{j}=\lambda _{j+1}=-\lambda _{j+2}$ . An unstable configuration of the $\lambda$ -field with one “defect” of size $\nu$ is defined as $-\lambda _{j-\nu -1}=\lambda _{j-\nu }=\lambda _{j-\nu +1}...=\lambda _{j+\nu }=-\lambda _{j+\nu + 1 }$. According to numerical calculations, a total energy of individual $\nu$ “defects” is greater than the energy of one “defect” of size $\nu$.

As an example, we present the calculations of the excitation energies in a chain with “one defect” as a function of the defect size $\nu$. Configurations with “defect” in an uniform configuration have energies lying in the gap, a number of excitations increases with increasing $\nu$ , so that for $1<\nu <5$ only one excitation is split off from the continuous spectrum, for $5<\nu <10$ , $10<\nu <15$ there are 2 and 3 such states, respectively (see in Fig. 6). We note, that the lattice with a double cell is formed by an uniform configuration of $\lambda$-field, and neither spin nor charge density waves are realized.

### Isotropic exchange interaction $J=K>0$

As noted above, in the absence of a magnetic field for an isotropic exchange interaction, the $\lambda$- and $\Lambda$-components of the ${Z}_2$-fields are equal and are solutions of Eq. (5). Along with this solution, there are also the number of non-trivial solutions: $\lambda \ne 0$ and $\Lambda =0$ , $\lambda =0$ and $\Lambda \ne 0$. Three solutions of Eq. (5) have the same energies, which follows from numerical calculations of the ground state energy as funsction of *J* at $h=0$ for different dimensions of the model. In the absence of an external magnetic field, the energies of the quasi-particle excitations are degenerate in spin. A magnetic field removes this degeneracy. For arbitrary values of magnetic field and isotropic exchange integral, a solution $\lambda \ne 0$, $\Lambda =0$ corresponds to a lower energy than a solution $\lambda = 0$, $\Lambda \ne 0$. Another nontrivial solution $\lambda \ne 0, \Lambda \ne 0$ not satisfy the self-consistent equations for $\lambda$ and $\Lambda$ for arbitrary *h*. KI is determined by the *XX*-exchange interaction (the value of *K* in Hamiltonian (Eq. 1)), the *ZZ*-exchange interaction (the value of *J* in Hamiltonian (Eq. 1)) does not participate in the formation of KI. Scattering processes with spin flip lead to the formation of KI in the Kondo lattice as it takes place in the Kondo problem.

## Conclusion

We studied the behavior of electron liquid in the spin-$\frac{1}{2}$ Kondo lattice at half-filling for different dimension. Due to antiferromagnetic exchange interaction between electrons and local moments a static $Z_2$- field is formed. An uniform configuration of the ${Z}_2$ -field corresponds to the ground state of KI, leads to formation of a lattice with a double cell. The spin or charge density waves are not realized in KI. In KI the spectrum of the quasi-particle excitations is symmetric about zero energy, as it takes place for the Majorana spectrum. At a critical value of a magnetic field, at which a gap closes, the phase transition to metal state is realized. The results of calculations largely coincide with the conclusions obtained by solving the one-dimensional Kondo lattice in the semiclassical approximation in the case of weak interaction in the continuum approximation^16^. According to^16^, KI forms due to strong antiferromagnetic fluctuations, the insulator state is realized in the case of the local doubling of period of the original lattice, the ground state in Kondo insulators is magnetically disordered. An exponentially small gap in^16^ is obtained in the proposed formalism in an one-dimensional lattice only when the branches of flat bands $\varepsilon_S$ are not taken into account in the calculations of $\lambda$ (Eq. 5). These branches are realized in a strong interaction, and it is not clear why they should disappear in a weak coupling. The value of the gap in the quasi-particle spectrum 60 meV, observed experimentally in *FeSi*^17^, is obtained from the above calculations.

## Data Availability

All data generated or analysed during this study are included in this published article. Correspondence and requests for materials should be addressed to I.N.K.
